# The efficacy and safety of remimazolam for the management of emergence agitation: a randomized, double-blind, placebo-controlled study

**DOI:** 10.3389/fmed.2025.1612779

**Published:** 2025-08-19

**Authors:** Xiaoxing Lu, Meiyan Zhou, Yao Lu, Jia Sun, Kexin Mao, Yangzi Zhu, Rongguo Wang, Yong Cao, Liwei Wang

**Affiliations:** ^1^Suzhou Medical College of Soochow University, Soochow, Jiangsu, China; ^2^Department of Anesthesiology, Southeast University Affiliated Xuzhou Central Hospital, Xuzhou, Jiangsu, China; ^3^Jiangsu Province Key Laboratory of Anesthesiology, Xuzhou Medical University, Xuzhou, Jiangsu, China; ^4^The Affiliated Xuzhou Clinical College of Xuzhou Medical University, Xuzhou, Jiangsu, China; ^5^Department of Anesthesiology, Xuzhou City Hospital of TCM, Xuzhou, Jiangsu, China

**Keywords:** remimazolam, emergency agitation, anesthesia recovery, sedation, otolaryngological surgery, randomized controlled trial

## Abstract

**Introduction:**

Emergence agitation (EA) is a common postoperative complication characterized by confusion, disorientation, and restless behavior that can lead to self-harm, the removal of medical devices, and other adverse events. This randomized, double-blind, placebo-controlled study was designed to assess the efficacy and safety of a novel benzodiazepine, remimazolam, in the management of EA.

**Methods:**

A total of 219 adults experienced EA (Riker Sedation-Agitation Scale SAS score ≥5) after otolaryngological surgery were randomly assigned (1:1:1 ratio) to receive one of the following three treatments: 2.5 mg remimazolam, 5.0 mg remimazolam, or placebo. The primary endpoint was the treatment success rate of EA, which was defined as an SAS score of <5 within 15 min after administration without the need for rescue sedation and no recurrence after 15 min. Secondary outcomes included rescue propofol dosage, EA duration, and the post-anesthesia care unit (PACU) discharge time. Adverse events were also monitored.

**Results:**

Both remimazolam groups (77.5% for 2.5 mg and 85.9% for 5.0 mg) had significantly higher treatment success rates compared to the placebo group (44.3%) (both *p* < 0.001). Additionally, they required less rescue propofol, had shorter EA durations, and faster PACU discharge times (all *p* < 0.001). Furthermore, the 2.5 mg group showed a lower incidence of hypoxia (7.0%) and hypotension (14.1%) compared to the placebo group (22.9% for hypoxia, 31.4% for hypotension) (*p* = 0.024 and 0.042, respectively). Exploratory analysis indicated that, for patients with dangerous agitation (SAS = 7), only the 5.0 mg group (83.3%) had a significantly higher treatment success rate than the placebo group (0%) (*p* < 0.001).

**Discussion:**

Our findings suggest that remimazolam is a promising option for managing EA in the PACU. For the entire study population, the 2.5 mg dose strikes an optimal balance between efficacy and safety. In patients with dangerous agitation, a 5.0 mg dose of remimazolam may offer potential benefits. These findings hold significant implications for guiding future therapeutic strategies for EA.

**Clinical trial registration:**

https://www.chictr.org.cn/, identifier ChiCTR2400085903.

## 1 Introduction

Emergence agitation (EA) is a postoperative complication frequently observed in patients recovering from general anesthesia, especially following the use of volatile anesthetics like sevoflurane (1–3). It is characterized by confusion, disorientation, and restless behavior that can lead to self-harm, the removal of medical devices, and other adverse events (4). The exact pathophysiological mechanism of EA remains unclear, and the brain’s mesolimbic dopaminergic reward circuitry may be involved in the pathological process of EA (5). The causes and risk factors of EA are varied and include pain, adverse stimuli (e.g., catheters, nasogastric tubes, and chest tubes), specific surgical procedures (such as otolaryngologic, oral, and abdominal surgeries), male gender, and the use of inhalation anesthetics (6–8). Based on these causes and risk factors, the incidence of EA varies widely, ranging from 10 to 80% (9–11). For instance, otolaryngological surgery has been associated with a high risk of EA in adults (1, 3). Although EA is usually self-limiting, addressing this issue is still critical, as the agitation and thrashing behavior of patients with EA can be hazardous for both the patients themselves and healthcare workers (12). In addition, EA is a strong predictor of postoperative delirium (POD), which results in prolonged hospital stays, increased healthcare costs, and higher risks of postoperative complications (7).

Several studies have focused on the prevention of EA (13–16), but only a few have specifically addressed its treatment (17, 18). The key focus in the treatment of EA is to achieve rapid and safe sedation. Remimazolam, a novel ultra-short-acting benzodiazepine (19), offers an alternative approach for managing EA. The pharmacokinetic profile of remimazolam allows for a rapid onset and offset of action, providing effective sedation with minimal respiratory depression (20). Studies have demonstrated the efficacy of remimazolam in various procedural sedations, including gastroscopy (21), colonoscopy (22), and bronchoscopy (23), with faster recovery times and fewer sedation-related complications (20, 24). However, it is currently unknown whether remimazolam can provide effective sedation for EA.

With EA remaining a challenge in anesthetic management, particularly in vulnerable populations, this randomized, double-blind, placebo-controlled aims to explore the efficacy and safety remimazolam in the treatment of EA in adult patients recovering from general anesthesia for otolaryngological surgery. We hypothesize that remimazolam can provide effective sedation for EA patients without increasing the risk of related complications such as respiratory and circulatory depression.

## 2 Materials and methods

### 2.1 Study population

This trial was conducted at Xuzhou Central Hospital, China. It was approved by the Institutional Ethics Committee of Xuzhou Central Hospital (XZXY-LK-20231017-0165) on October 17, 2023 and registered in the Chinese Clinical Trial Registry (registration number ChiCTR2400085903) on June 20, 2024. The trial protocol, statistical analysis plan, and related data are available from the corresponding author upon reasonable request. From January to June 2024, preparations for the implementation of this study were conducted, including pre-experimental work, consultations with experts, and the development of a patient recruitment plan. Patient enrollment officially took place from June 28, 2024 to December 31, 2024. All procedures adhered to the Declaration of Helsinki and written informed consent was obtained from all participants prior to enrollment. The Consolidated Standards of Reporting Trials (CONSORT) guidelines were followed when preparing this study. This trial included adult patients aged 18–65 years who were diagnosed with EA in the post-anesthesia care unit (PACU) following general anesthesia for otolaryngological surgery. Eligible participants had a BMI between 18.5 and 30 kg/m^2^ and an ASA physical status of I–III. Exclusion criteria were allergies to remimazolam or related drugs, cognitive or psychiatric disorders, difficult airways, surgeries lasting ≥4 h, pregnancy or breastfeeding, and recent substance abuse or participation in other clinical trials within 30 days.

The recruitment process for patients is divided into two phases: 1. Preliminary Screening (Day Before Surgery): on the day prior to surgery, a trained researcher conducted a preliminary screening of patients scheduled for otolaryngological procedures. The researcher assessed candidates based on predefined inclusion and exclusion criteria and obtained written informed consent. 2. Postoperative Assessment (Day of Surgery): After the surgery, two evaluators in PACU assessed the patients to diagnose the occurrence of EA. Patients who experienced EA were eventually enrolled in the trial, while those who did not experience it were excluded from the study.

### 2.2 Randomization and blind

This study was designed as a prospective, double-blind, randomized, parallel-group, exploratory trial. Randomization was performed by an independent statistician using a computer-generated randomization list, which was also maintained by him. Upon enrollment, the enrolled participants were randomly assigned in a 1:1:1 ratio to one of three groups: the 2.5 mg remimazolam group, the 5.0 mg remimazolam group, and the placebo group. The group assignments were concealed in sealed opaque envelopes. A trained PACU nurse opened the envelope and intravenously administered the trial medication. Solutions for all groups were prepared by a third-party pharmacist to ensure identical appearance and volume. Trained anesthesiologists evaluated the effectiveness of the treatment and collected the relevant data. The patients, investigators, and statisticians were blinded to the group assignments.

### 2.3 Study procedure

Following the completion of surgeries, the patients were admitted to the PACU. If any signs suggestive of emergence agitation (EA) appeared, two trained evaluators assessed the patient’s agitation status using the Riker Sedation-Agitation Scale (SAS) (Table S1). The scoring was as follows: 1–3—unarousable to sedated; 4—calm and cooperative; 5—agitated or mildly agitated; 6—very agitated; and 7—dangerous agitation. EA was defined as maximal SAS score ≥5 (4). Once diagnosed with EA, patients first received 50 mg of flurbiprofen as a baseline analgesia and were then randomly assigned to one of the following three treatments: 2.5 mg remimazolam (diluted in 5 mL normal saline), 5.0 mg remimazolam (diluted in 5 mL normal saline), or 5 mL normal saline. If the desired outcome (SAS < 5) was not achieved, half of the initial dose of the investigational medicinal product (IMP) was administered, with no more than five doses within 15-min, at intervals of ≥1 min. If the SAS score remained at 5 or higher after five trial doses or EA recurred 15 min later, treatment failure was declared, and 0.5 mg/kg of propofol was administered for rescue sedation. The dosage and administration of remimazolam in this study were based on the guidelines for mild to moderate sedation as specified in the remimazolam prescribing information (20, 24).

### 2.4 Anesthesia and perioperative management

Heart rate (HR), invasive blood pressure (IBP), electrocardiogram (ECG), oxygen saturation (SpO2), body temperature, and depth of anesthesia were continuously monitored throughout the procedure. The anesthesia protocol used in this study was total intravenous anesthesia (TIVA). Anesthesia was induced with intravenous administration of midazolam (0.05 mg/kg), etomidate (0.2 mg/kg), sufentanil (0.4 μg/kg), and cisatracurium (0.2 mg/kg). Anesthesia was maintained with propofol and remifentanil, targeting a bispectral index (BIS) between 40 and 60. At the conclusion of surgery, 20 μg of sufentanil and 4 mg of dexamethasone were administered for preemptive analgesia and to prevent postoperative nausea and vomiting, respectively. The tracheal catheter was removed once the patient could clearly respond to commands, had a tidal volume exceeding 6 mL/kg, and breathed normally. Patients were discharged from the PACU when the Modified Aldrete Score (Table S2) achieved 9 points. All participants were equipped with a patient-controlled intravenous analgesia (PCIA) device consisting of 2 μg/kg sufentanil in a total volume of 100 mL, with a continuous infusion rate of 2 mL/h for 48 h. Rescue analgesia with 50 mg of flurbiprofen axetil was administered if the Visual Analog Scale (VAS) pain score was ≥4.

### 2.5 Outcome measurements

The primary outcome was the success rate of EA management in the PACU. Successful treatment was defined as achieving a composite outcome (all three conditions must be met) of the following:

1. A reduction in the SAS score to below 5 within 15 min of the initial dose of IMP;
2. No requirement for rescue sedation;
3. No recurrence of EA 15 min after the first dose of the IMP.

Secondary outcomes included the total remimazolam dosage, amount of rescue propofol administered, duration of EA, and time to PACU discharge from the last dose of IMP or a remedial sedative.

Safety outcomes were assessed by tracking adverse events, including hypoxia (SpO2 < 90% or any event requiring intervention), heart rate abnormalities (sinus bradycardia: <40 bpm or ≥20% below baseline; sinus tachycardia: >100 bpm or ≥20% above baseline), blood pressure issues (hypertension: systolic ≥ 180 mmHg or diastolic ≥ 100 mmHg; hypotension: systolic ≤ 80 mmHg or diastolic ≤ 40 mmHg), and prolonged sedation (MOAA/S score ≤ 4 for over 60 min).

### 2.6 Statistical analysis

Sample size calculations were based on preliminary success rates for the placebo group and remimazolam groups (43.6% for the placebo group, 74.2% for the 2.5 mg remimazolam group, and 84.9% for the 5.0 mg remimazolam group). With a two-sided type I error rate of 0.05, a target power of 90%, PASS software (version15.0) determined that 58 participants per group were needed. To account for a 20% dropout rate, the sample size was adjusted to 73 participants per group.

Data analysis was performed using SPSS version 29.0 (SPSS Inc., Chicago, IL, USA). The Shapiro–Wilk test was used to assess the normality of continuous variables. Variables with normal distribution were expressed as mean (SD) and analyzed using one-way ANOVA, with Bonferroni post-hoc comparisons when significant. Non-normally distributed variables were presented as median (IQR) and evaluated using Kruskal-Wallis tests, with Bonferroni post-hoc comparisons when significant. Categorical variables were presented as numbers (proportions) and compared using Chi-square or Fisher’s exact tests. A two-sided *p* < 0.05 was considered indicative of a statistically significant difference. An exploratory *post hoc* analysis was conducted to evaluate the treatment effects of different doses of remimazolam in patients with varying levels of agitation.

## 3 Results

### 3.1 Demographics

**Figure 1:**
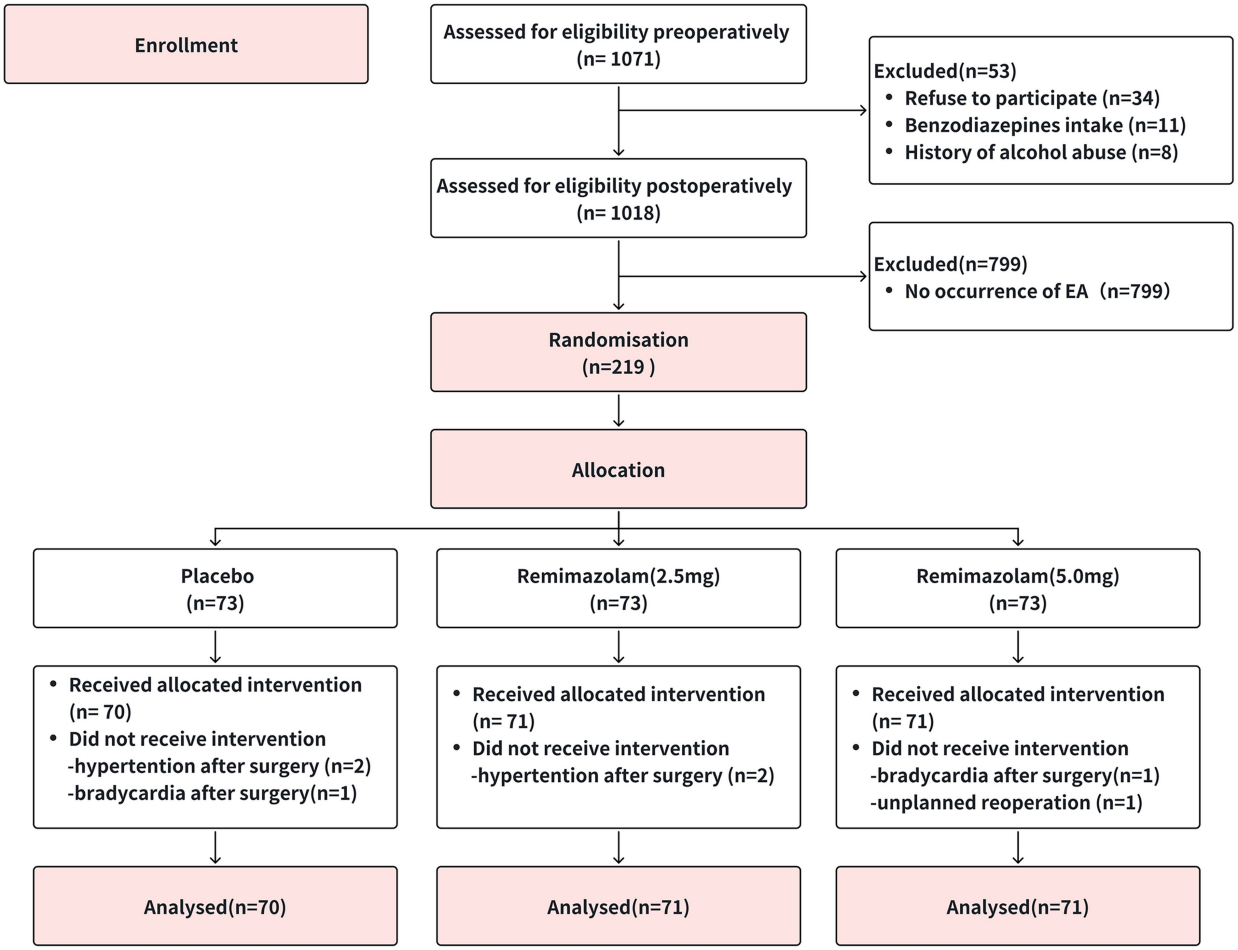
Flow diagram. A total of 219 patients were randomized, with 73 participants allocated to each group. Due to hemodynamic instability or unplanned reoperation, seven patients did not receive the intervention, resulting in 212 participants included in the final analysis.

A total of 1,071 participants were initially assessed for eligibility prior to surgery, resulting in the exclusion of 53 individuals. After surgery, 799 patients were further excluded from the study due to the absence of EA, while 219 patients who experienced EA were included in the study. Among these individuals, 113 participants (51.6%) were agitated (SAS = 5), 75 participants (34.2%) were very agitated (SAS = 6), and 31 participants (14.2%) were diagnosed with dangerous agitation (SAS = 7). The 219 participants were randomly assigned to three groups (1:1:1 ratio) (Figure 1). The baseline demographic and clinical characteristics of the participants were summarized (Table 1). Most of the participants were in their forties, with the majority being male. No significant differences were observed among groups in terms of the demographic data and clinical characteristics.

**Table 1:** Patient demographics and surgical characteristics Figure 1.

Characteristics	Placebo *n* = 70	Remimazolam (2.5 mg) *n* = 71	Remimazolam (5.0 mg) *n* = 71	*p* Value
Age (yr)	47.5 (32–54)	46 (35–56)	49 (36–57)	0.292
Sex				0.111
Male	45 (64.3%)	51 (71.8%)	39 (54.9%)	
Female	25 (35.7%)	20 (28.2%)	32 (45.1%)	
Height (cm)	170.37 ± 6.38	171.75 ± 7.10	170.28 ± 7.12	0.366
Weight (kg)	71.49 ± 8.98	72.28 ± 8.63	70.99 ± 8.83	0.097
BMI (kg/m^2^)	24.58 ± 2.31	24.45 ± 1.97	23.80 ± 1.97	0.061
ASA physical status				0.501
I	18 (25.7%)	11 (15.5%)	12 (16.9%)	
II	51 (72.9%)	57 (80.3%)	57 (80.3%)	
III	1 (1.4%)	3 (4.2%)	2 (2.8%)	
aCCI	1 (0–2)	1 (0–2)	1 (0–2)	0.160
APAIS score	11 (8–16)	12 (6–15)	10 (7–12)	0.151
Type of surgery				0.853
Ear surgery	10 (14.3%)	6 (8.5%)	8 (11.3%)	
Nasal surgery	21 (30%)	23 (32.4%)	24 (33.8%)	
Laryngopharynx surgery	39 (55.7%)	42 (59.2%)	39 (54.9%)	
Anesthesia duration (min)	50 (29.25–82)	45 (30–75)	54 (30–76)	0.533
Operation duration (min)	35 (15–70.75)	30 (20–60)	40 (20–60)	0.809
Indwelling urinary catheter	26 (37.1%)	21 (29.6%)	23 (32.4%)	0.628
Agitation status				0.943
Agitated (SAS = 5)	36 (51.4%)	37 (52.1%)	33 (46.5%)	
Very agitated (SAS = 6)	24 (34.3%)	25 (35.2%)	26 (36.6%)	
Dangerous agitation (SAS = 7)	10 (14.3%)	9 (12.7%)	12 (16.9%)	

Variables with normal distribution were expressed as mean ± (SD) and analyzed using one-way ANOVA. Non-normally distributed variables were presented as median (IQR) and evaluated using Kruskal-Wallis tests. Categorical variables were presented as numbers (proportions) and compared using Chi-square.

BMI, Body Mass Index; ASA, American Society of Anesthesiologists; aCCI, age-adjusted Charlson Comorbidity Index; APAIS, Amsterdam Preoperative Anxiety and Information Scale; SAS: Riker Sedation-Agitation Scale.

### 3.2 Primary outcomes

Treatment success rate was notably higher in the remimazolam groups (77.5% for 2.5 mg and 85.9% for 5.0 mg) compared to the placebo group (44.3%) (both *p* < 0.001). When comparing the remimazolam groups, the treatment success rate was slightly higher in the 5.0 mg group than in the 2.5 mg group, but there was no statistical significance (*p* = 0.579, Table 2). Within 15 min after the first dose, 87.3% participants in the remimazolam 2.5 mg group and 94.4% in the remimazolam 5.0 mg group and 85.7% in the placebo group achieved a reduction in SAS scores to below 5, with no statistically significant differences between the three groups. (*p* = 0.213, Table 2). However, compared to the placebo group (44.3%), more participants in the 2.5 mg (81.7%) and 5.0 mg (85.9%) remimazolam groups did not require remedial sedation (both *p* < 0.001, Table 2). Furthermore, the rates of no recurrence of EA in the remimazolam 2.5 mg and 5.0 mg were 85.9 and 90.1%, respectively, which were significantly higher than the 64.3% in the placebo group (both *p* < 0.01, Table 2).

**Table 2:** Achievement of primary efficacy end point (treatment success).

Efficacy end point	Placebo *n* = 70	Remimazolam (2.5 mg) *n* = 71	Remimazolam (5.0 mg) *n* = 71	*p* Value
Treatment success (composite end point)	31 (44.3%)	55 (77.5%)^a^	61 (85.9%)^a^	<0.001
SAS score dropped below 5 within 15 min after the first does of IMP	60 (85.7%)	62 (87.3%)	67 (94.4%)	0.213
No administration of rescue propofol	31 (44.3%)	58 (81.7%)^a^	61 (85.9%)^a^	<0.001
No recurrence of EA 15 min after the first does of IMP	45 (64.3%)	61 (85.9%)^a^	64 (90.1%)^a^	<0.001

Data are presented as number (%) and comparisons among groups were performed using Chi-square tests. If statistical differences were identified, pairwise comparisons were performed with Bonferroni correction to account for multiple testing. Compared with Group Placebo, ^a^*p* < 0.05. Compared with Group Remimazolam (2.5 mg), ^b^*p* < 0.05.

SAS, Riker Sedation-Agitation Scale; IMP, investigational medicinal product; EA, emergence agitation.

**Figure 2:**
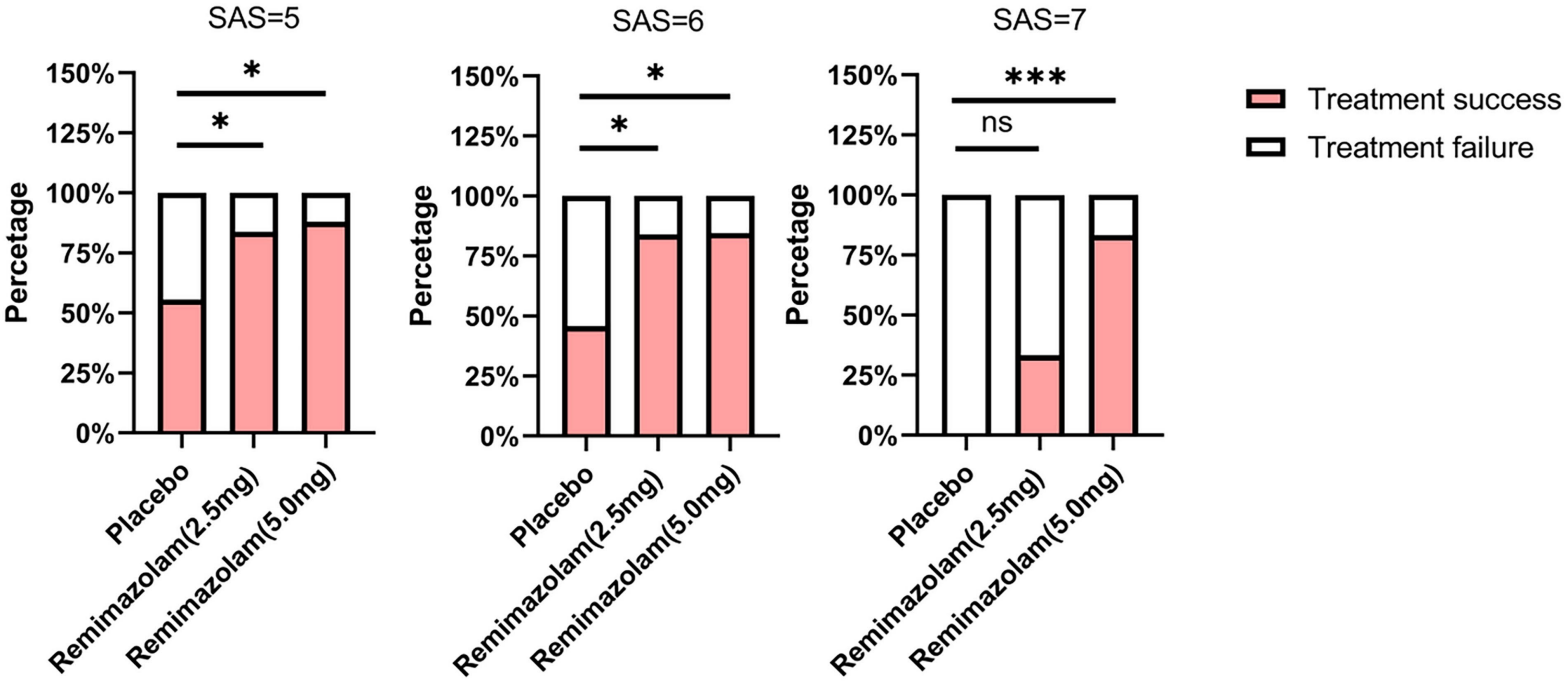
Comparison of treatment success at different levels of agitation. Comparisons among groups were performed using Chi-square tests. If statistical differences were identified, pairwise comparisons were performed with Bonferroni correction to account for multiple testing. Compared with Placebo group, *p < 0.05, ***p < 0.001. SAS, Riker Sedation-Agitation Scale.

In addition, we conducted an exploratory analysis of the primary endpoints. In patients with SAS scores of 5 or 6, both 2.5 mg and 5.0 mg remimazolam demonstrated a significantly higher EA resolution rate compared to the placebo group (*p* = 0.033 and 0.011 for SAS = 5, *p* = 0.022 and 0.020 for SAS = 6, respectively) (Figure 2). In patients with a SAS score of 7, the 5.0 mg dose of remimazolam (83.3%) improved the treatment success rate compared to the control group (0%) (*p* < 0.001), whereas the 2.5 mg dose (33.3%) did not (*p* = 0.260) (Figure 2).

### 3.3 Second outcomes

The total dosage of remimazolam was higher in the 5.0 mg group compared to the 2.5 mg group (5.00 [3.75–7.50] vs. 7.50 [5.00–10.00], *p* < 0.001). When comparing the total dosage of rescue propofol, both the 2.5 mg (0.00 [0.00–0.00]) and 5.0 mg remimazolam groups (0.00 [0.00–0.00]) had a minimal need for rescue propofol, whereas the placebo group (30.50 [0.00–42.13]) required a significantly higher amount (both *p* < 0.001). Both the 2.5 mg group (7 [4–10]) and the 5.0 mg remimazolam group (4 [2–7]) experienced significantly shorter EA durations compared to the placebo group (14 [11–19], both *p* < 0.001). Moreover, the time to discharge from the PACU was significantly shorter in both the 2.5 mg remimazolam group (38 [34–43]) and the 5.0 mg remimazolam group (46 [42–49]) than in the placebo group (54 [47–59], both *p* < 0.001). Notably, the 2.5 mg group had a significantly shorter discharge time than the 5.0 mg group (38 [34–43] vs. 46 [42–49], *p* < 0.001). (Table 3).

**Table 3:** Secondary outcomes.

Recovery quality parameter	Placebo *n* = 70	Remimazolam (2.5 mg) *n* = 71	Remimazolam (5.0 mg) *n* = 71	*p* Value
Total dosage of remimazolam, mg	–	5.00 (3.75–7.50)	7.50 (5.00–10.00)^b^	<0.001
Total dosage of rescue propofol, mg	30.50 (0.00–42.13)	0.00 (0.00–0.00)^a^	0.00 (0.00–0.00)^a^	<0.001
Duration of agitation, min	14 (11–19)	7 (4–10)^a^	4 (2–7)^a^	<0.001
Time to discharge from PACU from last dose of IMP/rescue, min	54 (47–59)	38 (34–43)^a^	46 (42–49)^ab^	<0.001

Data are presented as median (inter-quartile range) and comparisons among groups were performed using Kruskal-Wallis tests, followed by Bonferroni-adjusted pairwise comparisons. Compared with Group Placebo, ^a^*p* < 0.05. Compared with Group Remimazolam (2.5 mg), ^b^*p* < 0.05.

PACU, post-anesthesia care unit; IMP, investigational medicinal product.

### 3.4 Safety outcomes

Compared to the placebo group (22.9%), the incidence of hypoxia was significantly lower in the 2.5 mg remimazolam group (7.0%, *p* = 0.024), but not the 5.0 mg remimazolam group (15.5%, *p* = 0.798). Similarly, the 2.5 mg remimazolam group (14.1%, *p* = 0.042), but not the 5.0 mg group (22.5%, *p* = 0.702), showed a significantly lower incidence of hypotension compared to the placebo group (31.4%). Regarding other adverse events, including hypertension, tachycardia, nausea, vomiting, and prolonged sedation, there were no significant differences among the groups (Table 4).

**Table 4:** Incidence of treatment-emergent adverse event.

Adverse event	Placebo *n* = 70	Remimazolam (2.5 mg) *n* = 71	Remimazolam (5.0 mg) *n* = 71	*p* Value
Hypoxia	16 (22.9%)	5 (7.0%)^a^	11 (15.5%)	0.032
Hypotension	22 (31.4%)	10 (14.1%)^a^	16 (22.5%)	0.048
Hypertension	17 (24.3%)	13 (18.3%)	12 (16.9%)	0.506
Tachycardia	0 (0.0%)	0 (0.0%)	1 (1.4%)	1.000
Bradycardia	14 (20.0%)	5 (7.0%)	9 (12.7%)	0.075
Nausea	6 (8.6%)	6 (8.5%)	5 (7.0%)	0.933
Vomiting	1 (1.4%)	1 (1.4%)	2 (2.8%)	1.000
Prolonged sedation	2 (2.9%)	0 (0%)	0 (0%)	0.108

Data are presented as number (%) and comparisons among groups were performed using Chi-square tests or Fisher’s exact tests. If statistical differences were identified, pairwise comparisons were performed with Bonferroni correction to account for multiple testing. Compared with Placebo group, ^a^*p* < 0.05. Compared with Group Remimazolam (2.5 mg), ^b^*p* < 0.05.

## 4 Discussion

This trial was performed to evaluate the efficacy and safety remimazolam in the treatment of EA in adult patients recovering from TIVA for otolaryngological surgery. The major finding of our study was that both 2.5 mg and 5.0 mg remimazolam significantly improved treatment success rates compared to placebo. Remimazolam groups showed reduced need for rescue sedation and lower EA recurrence rates. The 5.0 mg dose was more effective in patients with dangerous agitation (SAS = 7). Both doses shortened EA duration and PACU discharge time compared to placebo, with the 2.5 mg dose leading to the fastest discharge. Safety analysis revealed that the 2.5 mg remimazolam group had significantly lower hypoxia and hypotension incidences than placebo, whereas the 5.0 mg group showed no significant differences. Other adverse events were similar across groups.

Numerous studies have been conducted on the prevention of EA, including both pharmacological and non-pharmacological approaches, such as dexmedetomidine, ketamine, and magnesium sulfate (25–27), and removing indwelling invasive devices as early as possible (4). Few clinical trials have evaluated the effects of therapeutic strategies for EA. A randomized controlled trial conducted in Germany evaluated the effects of physostigmine compared to a placebo in managing EA in preschool children who underwent intravenous anesthesia combined with sevoflurane. The study ultimately concluded that physostigmine did not demonstrate any significant therapeutic advantage in this context (28). A recent study conducted in China by Li et al. investigated the comparative effects of propofol and remifentanil in managing EA among adults undergoing inhalational anesthesia (sevoflurane) combined with intravenous sedation. This study found that the recurrence rate of EA within 15 min was significantly lower in the remifentanil group than in the propofol group (29.7% vs. 49.3%, *p* = 0.014). Furthermore, the use of propofol was associated with a significantly prolonged extubation time and an extended duration of stay in the PACU (18).

As a novel ultrashort-acting benzodiazepine, remimazolam can be titrated to achieve moderate levels and durations of sedation while maintaining a good safety profile (20, 29). A double-blind randomized controlled trial conducted in China demonstrated that the administration of remimazolam at the beginning of sutures effectively prevented emergence delirium in children undergoing laparoscopic surgery (9). Based on the dosing instructions in previous studies and the product label (20, 24), this study employed a titration method to evaluate the therapeutic effect of 2.5 mg and 5.0 mg doses of remimazolam on EA. Our study revealed that 21.5% of participants experienced emergence agitation (EA), which aligns with the incidence of EA (22.2%) reported by Hyo-Jin Kim in otolaryngological surgeries, where 88.6% of patients received TIVA (30). Moreover, both 2.5 mg and 5.0 mg doses of remimazolam achieved a high success rate in the treatment of EA compared with the placebo group. Although the 2.5 mg dose resulted in a slightly lower success rate for EA treatment than the 5.0 mg dose, the difference in the success rates between the two groups was not statistically significant.

In the placebo group, 44.3% achieved treatment success, which suggests that some patients can recover spontaneously without the need for sedative medication. This result was consistent with previous reports, indicating that EA is self-limiting (1). Further exploratory analysis revealed that in the placebo group, the self-recovered patients were mainly those with SAS scores of 5 or 6. However, patients with dangerous agitation, if not administered rescue sedation, may not have their symptoms alleviated. Both 2.5 mg and 5.0 mg remimazolam demonstrated good therapeutic effects in patients with mild to moderate agitation, nevertheless, in patients with dangerous agitation, only 5.0 mg remimazolam showed a significant therapeutic effect, suggesting that the dosage of remimazolam may need to be adjusted according to the severity of agitation. These findings were based on an exploratory analysis, and further conclusive results should be validated through adequately powered RCTs. Overall, our results provide valuable insights into optimal dosing strategies for managing EA in adult patients.

This study also investigated the quality of anesthesia recovery, with particular attention paid to the duration of EA, as well as the time of discharge from the PACU. Both remimazolam groups had significantly shortened EA duration, and patients in the 2.5 mg remimazolam group had the shortest time to discharge from the PACU. Furthermore, we conducted a rigorous monitoring process for treatment-emergent adverse events (TEAEs) such as hypoxia, hypotension, and bradycardia. In 2019, a multicenter US study showed that in adults undergoing bronchoscopy, remimazolam, with fentanyl for analgesia and midazolam for rescue sedation, does not increase hypoxemia or arrhythmia risks compared to placebo and significantly reduces hypotension incidence (20). A recent meta-analysis involving 4,516 adults found that remimazolam, compared to propofol during gastrointestinal endoscopy, was associated with significantly lower rates of respiratory depression, hypotension, treatment-requiring hypotension, and bradycardia (31). The present study demonstrated that the incidence of hypoxia and hypotension was lower in the 2.5 mg remimazolam group than in the placebo group, where a higher number of patients required larger doses of propofol for rescue sedation. However, this difference was not observed in the 5.0 mg remimazolam group, indicating that remimazolam (2.5 mg) may offer a more favorable safety profile for managing EA. Additionally, no statistically significant differences were observed in hypertension, tachycardia, nausea, vomiting, or prolonged sedation among the three groups. The comprehensive nature of our data collection and the focus on both efficacy and safety outcomes provides a more complete understanding of the role of remimazolam in postoperative sedation management.

The placebo-controlled design rigorously evaluated the independent efficacy of remimazolam in managing EA, which minimized potential confounding variables and established a clearer causal relationship between the intervention and therapeutic outcomes. Additionally, recognizing that EA is generally a self-limiting condition (1), the results from the placebo group provided valuable insights into the spontaneous recovery patterns of patients with varying SAS scores. However, the absence of non-inferiority or superiority tests using established pharmacological interventions such as propofol as a control group hampers the ability to obtain direct comparative efficacy analyses between remimazolam and these established therapies. Future research should address this gap and build a more comprehensive evidence base for optimal pharmacotherapy in the management of EA.

Our study had several other limitations. First, it was conducted at a single center, which may introduce bias due to variations in clinical practice and patient selection. To improve the generalizability of our findings, multicenter and international studies are necessary. Secondly, the patients included in this study were those receiving ENT surgeries under TIVA, the effectiveness of remimazolam for patients with other anesthesia methods and types of surgeries needs further validation. Additionally, the follow-up period was limited to 24 h postoperatively, potentially missing long-term adverse effects or delayed recovery outcomes. Finally, the study did not include enough patients with dangerous agitation to thoroughly assess the efficacy and safety of 5.0 mg remimazolam in this subgroup. Future research should examine the relationship between agitation severity and remimazolam dosage to identify the most effective and safe dosing strategy for patients with varying levels of agitation. Additionally, extending the follow-up period could provide valuable insights into the long-term safety and recovery profiles of remimazolam for the management of EA.

## 5 Conclusion

Our study demonstrates that remimazolam is a promising option for managing EA in the PACU. The 2.5 mg dose offers an optimal balance of efficacy and safety, effectively addressing EA and minimizing side effects for most patients. For those experiencing severe agitation, a 5.0 mg dose may provide additional benefits, enabling rapid sedation without significantly increasing adverse risks. This dosing flexibility allows clinicians to tailor treatments to individual patient needs, improving comfort and recovery. Incorporating remimazolam into PACU protocols has the potential to enhance patient outcomes, reduce recovery times, and alleviate nursing workloads. Overall, our findings support further exploration of remimazolam as a standard treatment for EA in post-operative settings.

## Data Availability

The raw data supporting the conclusions of this article will be made available by the authors without undue reservation.
